# Memory-Related Limitations and Care Needs of Gender Minority BIPOC Adults

**DOI:** 10.1093/geroni/igab046.1565

**Published:** 2021-12-17

**Authors:** Ethan Cicero, Nickolas Lambrou, Whitney Wharton, Jason Flatt

**Affiliations:** 1 Emory University School of Nursing, Atlanta, Georgia, United States; 2 University of Wisconsin, Madison, Madison, Wisconsin, United States; 3 Emory University, Atlanta, Georgia, United States; 4 University of Nevada Las Vegas, Las Vegas, Nevada, United States

## Abstract

Limited research exists investigating cognitive impairment and Alzheimer’s disease and related dementias (ADRD) among gender minority (GM) adults (transgender/non-binary/, including examining memory-related challenges among GMs who also identify as Black, Indigenous, or People of Color (BIPOC). 2015-2019 Behavioral Risk Factor Surveillance System data were used to explore care needs of GM and cisgender (CG) adults with subjective cognitive decline (SCD, N=441), which may be the first clinical manifestations of ADRD. Regression models examined SCD-associated functional limitations and care needs among GM-BIPOC, GM-White, CG-BIPOC, and CG-White adults. GM-BIPOC and GM-White were 2-4x more likely to have SCD-related limitations, require assistance with daily tasks, be unable to do day-to-day or social activities when compared to CG-White. GM-BIPOC were 2-5x more likely to be uninsured and experience cost-related healthcare barriers compared to GM-White and CG-White/BIPOC. Additional research is needed to improve care and well-being for this understudied population.

